# Le pseudokyste du pancréas chez l’enfant: à propos de 7 cas

**DOI:** 10.11604/pamj.2019.32.77.14688

**Published:** 2019-02-12

**Authors:** Hind Cherrabi, Hind Aboueljaoud, Abdoulaye Diallo Harouna, Aziz El Madi, Khalid Khattala, Youssef Bouabdallah

**Affiliations:** 1 Service de Chirurgie Pédiatrique, Hôpital Mère Enfant, CHU Hassan II, Faculté de Médecine et de Pharmacie de Fès, Université Sidi Mohammed Ben Abdellah, Fès, Maroc

**Keywords:** Pseudokyste, enfant, surveillance, traitement endoscopique, traitement chirurgical, Pseudocyst, child, monitoring, endoscopic treatment, surgical treatment

## Abstract

Le pseudokyste du pancréas est une affection bénigne, rare, en particulier chez l'enfant. Ce sont des collections de suc pancréatique, dépourvues de revêtement épithélial, et dont la localisation peut être intra ou extra pancréatique. Nous rapportons une étude rétrospective de 7 enfants colligés au service de chirurgie pédiatrique du centre hospitalier universitaire Hassan II de Fès, durant une période de 11 ans, allant du 1^er^janvier 2005 au 31 décembre 2016. Tous les enfants étaient de sexe masculin, l'âge moyen était de 6,6 ans (15 mois-12 ans). Un antécédent de traumatisme abdominal était retrouvé dans 4 cas, le ballonnement abdominal, la douleur, les vomissements et les troubles du transit étaient les principaux motifs de consultation. L'examen clinique retrouvait chez tous les enfants une sensibilité épigastrique et une altération de l'état général, l'échographie abdominale et le scanner abdominal réalisés respectivement ont permis de poser le diagnostic de pseudo kyste du pancréas avant l'intervention chirurgicale. Six malades sur sept ont été opérés ; Nous avons opté pour la dérivation interne (anastomose gastro-kystique) dans 4 cas, une dérivation externe dans 2 cas, et l'abstention thérapeutique dans le dernier cas. Les Pseudo kystes du pancréas sont des affections rares, néanmoins, ils sont de loin les plus fréquents (80% des lésions kystiques du pancréas étant des pseudo kystes, et sont la conséquence d'une pancréatite aigüe et/ou chronique, d'un traumatisme pancréatique, ou d'une obstruction canalaire pancréatique.

## Abstract (en)

Pancreatic pseudocyst is a rare benign condition, in particular among children. It is an intra or extra pancreatic juice collection lacking of an epithelial lining. We conducted a retrospective study of 7 children whose medical record data were collected in the Department of Paediatric Surgery at the University Hospital Hassan II in Fez, over a period of 11 years, from 1 January 2005 to 31 December 2016. All children were male, their average age was 6.6 years (15 months - 12 years). An history of abdominal trauma was found in 4 cases, abdominal bloating, pain, vomiting and transit disorders were the primary reason for consultation. Clinical examination showed epigastric tenderness and an alteration of the general state in all cases. Abdominal ultrasound as well as abdominal CT scan helped to diagnose pancreatic pseudocyst before surgery. Six patients, out of a total of seven, underwent surgery; we opted for internal bypass (gastro-cystic anastomosis) in 4 cases, external bypass in 2 cases and therapeutic abstention in one case. Pancreatic pseudocysts are rare. Nevertheless, they are the most frequent (80% of pancreatic cystic lesions are pseudocysts and due to acute and/or chronic pancreatitis, pancreatic trauma or pancreatic ductal obstruction).

## Introduction

On définit habituellement les pseudo-kystes du pancréas comme « des collections liquidiennes riches en amylases sans paroi propre, dépourvues de revêtement épithélial, qui résultent des remaniements de foyers de nécrose, de siège intra ou extra pancréatique. Ils apparaissent 4 à 6 semaines après le début de l'épisode aigu d'une pancréatite, compliquent une pancréatite chronique ou encore font suite à un traumatisme abdominal. » Le diagnostic de cette pathologie, difficile autrefois est actuellement aisé grâce aux progrès de l'imagerie médicale et notamment après l'avènement de l'ultrasonographie, la tomodensitométrie et la cholangio-pancréato-graphie rétrograde endoscopique. Leur prise en charge est complexe, et les discussions divergent entre les auteurs, concernant notamment le choix du moment idéal de l'intervention après la découverte du pseudokyste, et le type de procédé à utiliser offrant les meilleurs résultats.

## Méthodes

Notre travail propose une étude rétrospective de 7 cas colligés au service de chirurgie pédiatrique du centre hospitalier universitaire Hassan II de Fès, durant une période de 11 ans, allant du 1^er^ Janvier 2005 au 31 Décembre 2016. L'objectif de ce travail est d'élucider les propriétés épidémiologiques, cliniques, et paracliniques caractérisant cette pathologie.

## Résultats

Il s'agit de 7 garçons dont l'âge moyen était de 6,6 ans avec des extrêmes allant de 15 mois à 12 ans. Parmi les 7 patients, 2 cas ont été victimes d'une chute avec coup de guidon de bicyclette (28%); chute avec réception sur l'abdomen dans 4 autres cas (57%) et le cinquième enfant avait une pancréatite aigüe d'origine idiopathique (14%) qui s'est compliquée lors de sa surveillance d'un pseudokyste du pancréas. Leur répartition anatomique se faisait ainsi: 4 se trouvaient au niveau du corps du pancréas (soit 57%); 2 au niveau de la jonction corporéocaudale (soit 28%) ; Et 1 au niveau de l'isthme du pancréas (soit 28 %). La douleur épigastrique est le signe clinique révélateur majeur et prédominant, retrouvé chez tous les patients dans notre étude (soit 100% des cas) volontiers intense, de siège épigastrique et à irradiation dorsale, post prandiale précoce. Elle s'accompagne dans tous les cas de nausées et vomissements. Une fièvre aux alentours de 38° a été notée chez 2 patients soit (28% des cas) L'altération de l'état général a été rapportée par tous nos patients. L'examen clinique a montré une sensibilité épigastrique chez 5 patients (71%), et une sensibilité généralisée chez les deux autres (28%). La palpation abdominale a objectivé une masse épigastrique chez 3 malades soit (42% des cas). Une matité des flancs a été objectivée chez tous nos patients soit (100%). Bilan biologique: 1) numération Formule Sanguine (NFS): tous les patients de notre série en ont bénéficié objectivant une anémie hypochrome microcytaire dans 2 cas soit (28%) qui n'a pas nécessité de transfusion; 2) la lipasémie était constamment élevée allant de 5 à 30 fois la normale ; 3) une hyperamylasémie a été objectivée chez tous nos patients allant de 3 à 15 fois la normale; 4) CRP: un enfant avait un syndrome inflammatoire biologique avec une CRP élevée à 108 mg/l.

**Bilan radiologique:** 1) abdomen sans préparation réalisé chez tous nos patients, s'est révélé normale dans 100% des cas ; 2) radiographie thoraciqueréalisée chez tous les patients et s'est révélée normale chez tous les patients; 3) échographie abdominale: elle a été réalisée chez tous les patients et a permis d'objectiver un épanchement péritonéal de moyenne abondance chez tous nos patients (100% des cas) et de retrouver 1 PKP chez 2 patients (28% des cas) et deux PKP chez 1 autre patient (14%); 4) tomodensitométrie abdominale: tous les patients ont bénéficié d'au moins un examen tomodensitométrique abdominal, elle a confirmé dans tous les cas la présence de PKP: + La TDM abdominale initiale a montré une lésion linéaire de la jonction corporéo-caudale dans 2 cas (28%), une fracture isthmique du pancréas dans un cas (14%), un épanchement de moyenne abondance sans lésion viscérale décelable dans deux cas (28%) et un pancréas augmenté de taille dans un cas (14%); + le contrôle scannographique à J 10 a montré un pseudokyste du pancréas chez 5 enfants mesurant respectivement 12, 11 et 2,6 cm de grande axe. Ce n´est qu'au contrôle de J30 qu´on découvre le pseudokyste chez les deux autres patients; 5) l'IRM abdominale, aucun cas n'a bénéficié d'une IRM dans notre étude; 6) CPRE: elle n'a pas été réalisée dans notre série

**Le traitement:** 1) l'abstention thérapeutique avec surveillance chez l'enfant dont le pseudokyste mesurait 26 mm (14% des cas) et qui a été suffisante; 2) un drainage externe chez 2 patients (28% des cas) dont la surveillance n'avait pas contribué à la guérison et chez qui on a suspecté une rupture du pseudokyste vu l'aggravation de la symptomatologie clinique et de l´épanchement intra-péritonéale; 3) l'anastomose gastro-kystique a été pratiquée chez 4 patients (60%).

**Evolution:** 1) les suites postopératoires étaient simples chez tous nos patients; 2) l'alimentation entérale pauvre en lipides a été reprise progressivement à J10; 3) le drain s´est tari à J15 pour l´enfant qui a bénéficié d'un drainage externe; 4) par ailleurs un patient a été opéré 5 mois après pour syndrome de jonction pyélo-urétérale découvert fortuitement lors de son hospitalisation pour la pancréatite aigüe; 5) aucune mortalité ni morbidité postopératoire n'ont été notées dans notre série; 6) on a noté une disparition clinique et radiologique du PKP dans 80% des cas de notre série, qu'ils soient opérés ou simplement surveillés.

## Discussion

**Figure 1:**
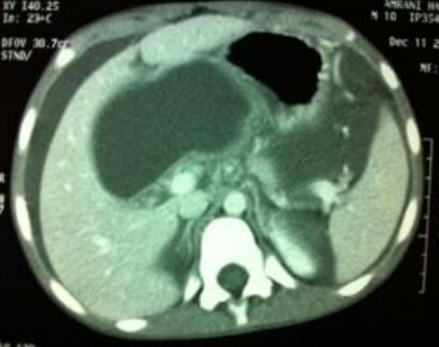
Image scannographique d’un pseudo kyste en maturation

**Figure 2:**
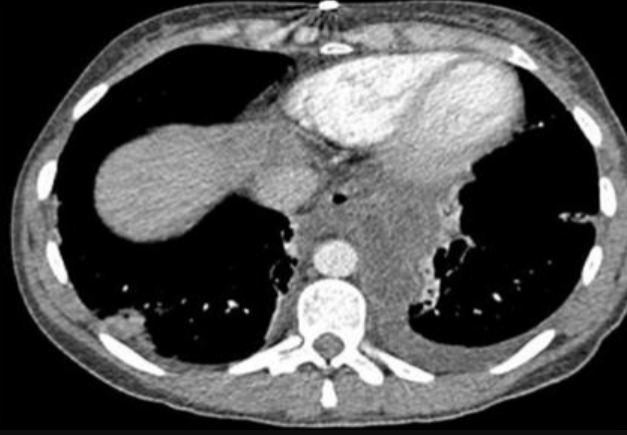
Image scannographique montrant l’extension intra médiastinale du pseudokyste de pancreas

Cette affection se voit à tout âge, le plus jeune malade porteur d'un faux kyste du pancréas est une fillette âgée de 3 mois et a été rapportée par Stone *et al.* [1] en 1967. L'âge moyen de la plupart des séries pédiatriques publiées est entre 6 et 7,5 ans [2, 3]. Les pseudo-kystes du pancréas (PKP) est une pathologie rare chez l'enfant [4]. Si les pseudokystes chez l´adulte font partie des complications des pancréatites chroniques dans 20-80% des cas selon les séries surtout d´origine éthylique, les traumatismes abdominaux fermés restent l´étiologie la plus fréquente des PKP de l´enfant [5-7]. Les pancréatites aigue viennent au deuxième rang [8]. Donc, on distingue en chirurgie pédiatrique deux formes pathogéniques de pancréatites: traumatique ou non traumatique. Les signes cliniques sont dominés par la douleur et la présence de la masse abdominale, la fièvre pouvant être en rapport avec avec une surinfection du kyste. L'échographie Doppler n´a que peu de place dans le diagnostic positif de pseudokyste, car elle est très inférieure au scanner pour le bilan d´extension lésionnelle et pour le diagnostic différentiel avec les autres formations tumorales kystiques [9]. Selon SPIEGELMAN, la TDM aide à différencier les collections liquidiennes des pseudokystes vrais. Une collection liquidienne aigue ne possède pas de paroi bien définie [10]; on a alors estimé un temps de 4 semaines suffisant à la maturation d'une collection liquidienne aigue pour devenir un pseudokyste (Figure 1, Figure 2). Le scanner comporte toutefois certaines limitations : outre l'irradiation, il nécessite l'injection de produit de contraste iodé dont les conséquences sur la fonction rénale ne doivent pas être négligées. Il analyse mal le contenu des pseudokystes et est peu performant pour l´analyse des communications éventuelles entre conduit pancréatique et pseudokyste. L'histoire clinique du patient est d'une importance capitale, si un patient n'a pas d'antécédents de pancréatite et qui présente une collection pancréatique, un diagnostic autre que le PKP doit être évoqué [11]. L'échographie et la TDM abdominales permettent la réalisation de ponctions exploratrices guidées dont l'apport est d'un grand intérêt [11]. Les performances de la cholangio-pancréato-IRM permettent en outre une meilleure analyse des rapports du kyste avec les conduits pancréatiques et des communications entre ces structures. L'IRM pancréatique a donc comme intérêt [12]: 1) d'avoir une valeur diagnostique probablement équivalente à celle du scanner. 2) Mettre mieux en évidence des débris solides dans une collection péripancréatique. Il s'agit d'une méthode invasive. Elle permet seulement de faire une opacification des voies biliaires intra et extra-hépatiques et du canal de Wirsung. Elle ne permet cependant pas de réaliser un geste thérapeutique [13].

**Figure 3:**
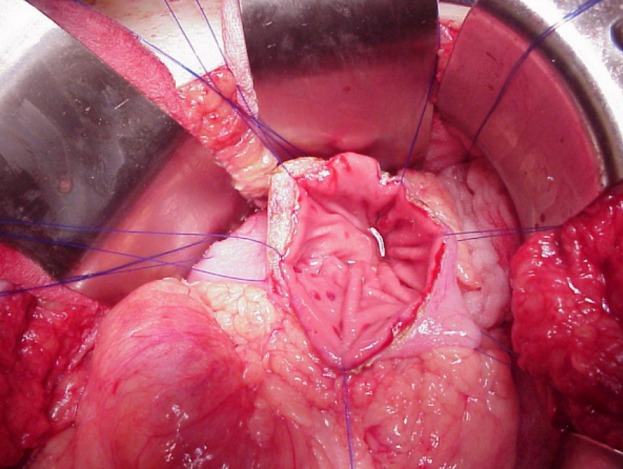
Image peropératoire montrant la gastrectomie antérieure

**Figure 4:**
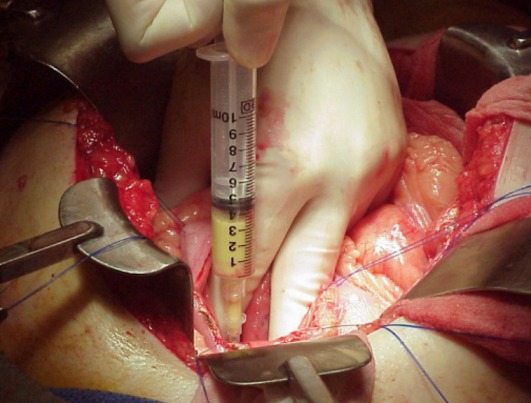
Image peropératoire montrant la collection liquidienne contenait des débris nécrotiques. Ce contenu est aspiré et adressé au laboratoire pour analyse

**Figure 5:**
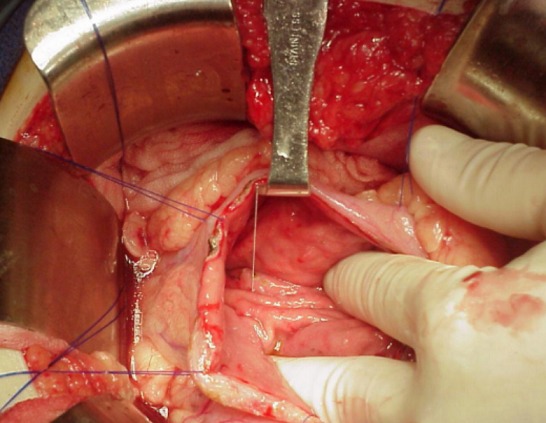
Un fil guide est introduit par l’intermédiaire d’une aiguilles pour aider à la pratique de la kysto-gastrostomie

**Figure 6:**
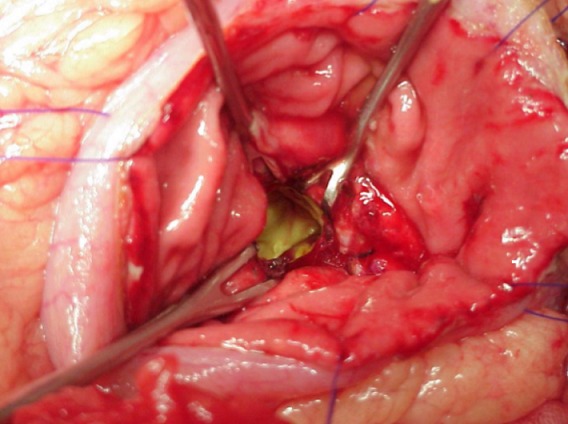
Kystogastrostomie terminée chez un patient souffrant d’un PKP. Une portion de la paroi du PKP doit être excisée et envoyée pour étude anatomopathologique afin de confirmer la nature non épithéliale de la paroi. La paroi postérieure de l’estomac et la capsule du PKP sont suturé ensemble

Si l´évolution des collections aigues de la pancréatite se fait le plus souvent vers la résolution spontanée, avec la persistance de pseudokystes dans uniquement 5 à 37% des cas, l´évolution spontanée des pseudokystes est plus partagée et plus difficile à prévoir. Si l´on considère les pseudokystes de plus de 6 cm de diamètre, persistants plus de 6 semaines après le début de l´épisode aigu de pancréatite, 50% évolueront vers une forme symptomatique ou compliquée, alors que 50% demeureront asymptomatiques ou disparaitront spontanément [14]. Même dans le cas de pseudokystes de grande taille (supérieur à 10 cm), il n´existe pas de différence en termes de morbidité, mortalité ou récidive post-traitement comparativement aux kystes de plus petite taille (58 mm). En fait dans la littérature, aucun facteur étiologique ou morphologique n´est prédictif de l´évolution d´un pseudokyste. Les résultats du traitement conservateur sont meilleurs chez l'enfant que chez l'adulte, surtout dans les cas de PKP faisant moins de 5 cm de diamètre. La surveillance clinique, biologique et radiologique est la règle. Le drainage percutané guidé par imagerie est pour certains auteurs la méthode la plus fiable, au point d'être la technique de première intention de traitement des PKP [15-18]. Les résultats des traitements endoscopiques et chirurgicaux sont comparables en termes d'efficacité, mais avec un coût moindre, une plus courte durée d'hospitalisation et une meilleure qualité de vie après le drainage endoscopique [19]. La mortalité liée au traitement est légèrement moindre avec le traitement endoscopique (0,2 % vs 2,5 %) [19]. Le drainage transpapillaire, lorsqu'il est techniquement et anatomiquement possible, semble être la technique la plus sûre avec un taux excellent de succès estimé à 60- 95% et un taux de récidive à 2 ans faible estimé à 0- 15% [20]. La kystogastrostomie par laparoscopie est une technique nouvelle, encore assez peu développée. La plupart des articles sur le sujet concernent des cas cliniques [21] ou des courtes séries [22]. Face au développement de ces techniques endoscopiques et radiologiques, les indications chirurgicales sont en nette régression. Les indications du drainage externe se résument aux PKP infectés à parois mal organisées après échec d'un traitement percutané ou certains PKP hémorragiques après échec de l'embolisation si une résection n'est pas envisageable en raison de l'état du malade ou des conditions locales [23]. les dérivations kysto-digestives (Figure 3, Figure 4, Figure 5, Figure 6) représentent d'après la plupart des auteurs les meilleures techniques chirurgicales de dérivation des PKP [24-26] les résections chirurgicales sont exceptionnelles.

## Conclusion

Les pseudokystes du pancréas est une pathologie rare chez l'enfant. Le diagnostic se fait sous un faisceau d'arguments cliniques biologiques et radiologiques. La décision thérapeutique dépend de la taille et le retentissement. Le traitement endoscopique semble plus intéressant avec de bons résultats.

### Etat des connaissances actuelles sur le sujet

- Les Pseudo Kystes du pancréas sont considérés comme une entité rare en pédiatrie;
- La particularité en est le faite qu'ils surviennt le olus souvent dans un contexte post traumatique tandis que chez les adultes,la cause est majoritairement lithiasique;
- Leur prise en charge est complexe, et les discussions divergent entre les auteurs, concernant notamment le choix du moment idéal après la découverte du pseudokyste, et le type de procédé à utiliser offrant les meilleurs résultats.

### Contribution de notre étude à la connaissance

- Elucider les propriétés épidémiologiques, cliniques, et paracliniques caractérisant cette pathologie;
- Exposer les modalités thérapeutiques;
- Discuter la variabilité de la prise en charge thérapeutique selon les équipes et dans notre série avec une stratégie codifiée.

## Conflits d’intérêts

Les auteurs ne déclarent aucun conflits d’intérêts.
